# Breastfeeding practices on postnatal wards in urban and rural areas of the Deyang region, Sichuan province of China

**DOI:** 10.1186/s13006-016-0070-0

**Published:** 2016-05-14

**Authors:** Haoyue Gao, Qi Wang, Elizabeth Hormann, Wolfgang Stuetz, Caroline Stiller, Hans Konrad Biesalski, Veronika Scherbaum

**Affiliations:** Institute of Social Science in Agriculture (430b), University of Hohenheim, Museumsfluegel, Stuttgart, 70599 Germany; Medical Society of Deyang City, Sichuan, Public Health Bureau of Deyang, Lushan Nan Road No. 299, Jingyang District, Deyang, 618000 China; Europäisches Institut für Stillen und Laktation, Wittberg 14, Kramsach, 6233 Austria; Institute of Biological Chemistry and Nutrition (140a), University of Hohenheim, Garbenstraße 30, Stuttgart, 70599 Germany; Food Security Center (793), University of Hohenheim, Wollgrasweg 43, Stuttgart, 70599 Germany

**Keywords:** Breastfeeding, Exclusive breastfeeding, Birth outcomes, Local belief

## Abstract

**Background:**

Despite the efforts that have been made to promote breastfeeding in China since the 1990s, there is still a very low prevalence of exclusive breastfeeding. The objective of this study was to assess the current situation of infant feeding practices during the postpartum hospital stay in urban and rural areas of the Deyang region.

**Methods:**

Cross-sectional sampling was used in two urban hospitals and five rural clinics in the Deyang region of southwestern China. Interviews with mothers after delivery (urban *n* = 102, rural *n* = 99) were conducted before discharge and five focus group discussions were held.

**Results:**

The prevalence of Caesarean section was high in both urban and rural areas (63.9 % urban vs. 68.4 % rural). After birth, nearly all mothers (98.0 % urban vs. 99.0 % rural) initiated breastfeeding. One week after delivery, the prevalence of exclusive breastfeeding was 8.0 % (9.8 % urban vs. 6.1 % rural), almost exclusive breastfeeding 34.5 % (29.4 % urban vs. 39.8 % rural), mixed feeding 56.0 % (58.8 % urban vs. 53.1 % rural), and exclusive formula feeding 1.5 % (2.0 % urban vs. 1.0 % rural).

Breastfeeding initiation (≤ two days after birth) was positively associated (Odds Ratio [OR] 1.97, 95 % Confidence Interval [CI] 1.11, 3.50) with exclusive and almost exclusive breastfeeding, whereas birth length under 50 cm (OR 0.48, 95 % CI 0.26, 0.87), mother’s education > 12 years (OR 0.46, 95 % CI 0.24, 0.88) and mother’s lack of knowledge about the importance of colostrum (OR 0.35, 95 % CI 0.14, 0.86) were negatively associated with almost exclusive breastfeeding.

**Conclusion:**

Although disparities between urban and rural areas exist, the situation of infant feeding is inadequate in both settings. The high prevalence of Caesarean section, the mothers’ poor knowledge of the physiology of breast milk production, the mothers’ lack of breastfeeding confidence, the widespread advertising of breast milk substitutes, and the changing perception of the function of breasts, may influence the unfavorable breastfeeding behavior observed in the study area.

## Background

Policymakers in China have become aware of the importance of breastfeeding and have set clearly defined goals for the promotion of breastfeeding in *The National Program of Action for Child Development in China* [1–3] (infant breastfeeding of 80 % by 2000 and promoting exclusive breastfeeding [1]; infant breastfeeding of 85 % by 2010 [2]; exclusive breastfeeding up to six months to reach 50 % by 2020 [3]). Despite many efforts that have been made to promote breastfeeding in China since the 1990s [4–7], the actual situation is, nevertheless, lagging far behind the proposed goals.

One cohort study in northwest China in 2007–2010 [8] showed that 96 % of the mothers with newborns initiated breastfeeding. The prevalence of exclusive breastfeeding was 24 % at 14 days after birth and 3 % at six months of age [8]. The prevalence of any breastfeeding at six months, 12 months and 24 months of age was 70, 30 and 2 % respectively [8]. In 2010, a breastfeeding study of 2354 children, conducted in central and western China (including Sichuan province), indicated that 29 % of infants under six months were exclusively breastfed, while 56 % of children at one year and 9 % of children at two years of age received their mothers’ milk in addition to complementary feeding [9]. One special report by Harney about infant formula sales in China pointed out that the prevalence of exclusive breastfeeding at six months of age could be as low as 0.2 % in parts of China [10]. The national breastfeeding data in China (data from UNICEF, 2012–2014), shows the prevalence of exclusive breastfeeding under six months was 28 % with no increase over the last three years [11–13]. Meanwhile, information about the proportion of young children who were breastfed up to two years of age is still not available [11–13]. Alongside the low prevalence of exclusive breastfeeding and any breastfeeding in China in 2012, China had already become the world’s largest infant formula market with a marketing scale of 38.5 billion RMB (Chinese currency, 6.2 billion US dollars). The number of newborns was 16 million in 2012 [10, 14].

In 2015, the Chinese government published a new population policy stating that “all resident couples will now be allowed to have two children” [15]. This was the most important reformation of the one-child policy since 1979 [16]. In order to meet the challenges of the next peak birth period in China brought about by the new “two child policy”, and to reduce infant mortality and improve young children’s health, it is recommended that breastfeeding, rather than infant formula, should be promoted more vigorously [17, 18].

In Sichuan province, nearly 60 % of the people live in rural areas [19]. Recently, one study indicated that, in 2012 the mortality rate of infants (<1 year, per 1000 live births) in rural areas was more than double that in urban areas (5.2 in urban vs. 12.4 in rural) [20]. Malnutrition is closely correlated with child mortality [21], which could be prevented by optimal feeding. Many other Chinese studies have shown similar findings as the growth of rural infants and children was behind that of children living in urban areas [21–24], while poor infant feeding practices (including suboptimal breastfeeding) were common in both areas [25–28]. One study carried out in Sichuan determined that poor feeding methods, rather than limited food resources, caused the disparity between urban and rural children. Thus, culturally appropriate interventions are needed to close these health gaps between urban and rural children [23]. Until recently, limited data about feeding practices and related local beliefs had been published in the study area. Nevertheless, the feeding practice at the beginning of a newborn’s life is one of the key factors for successful breastfeeding. Consequently, the objective of this study became to assess the current situation of infant feeding practices in urban and rural areas of the Deyang region as the basis for the development of adequate nutrition and health education messages.

## Methods

### Infant feeding definitions

In this study, the following infant feeding definitions were used:

- Exclusive breastfeeding (EBF): Breastfeeding only, with no other food or liquid, or water, with the exception of drops or syrups consisting of vitamins, mineral supplements or medicine. This means that the infant did not receive any type of prelacteal food and no supplementary food.
- Almost exclusive breastfeeding (AEBF): The infant received prelacteal food before the onset of breastfeeding, but no supplementary food was offered after breastfeeding was established [29].
- Mixed feeding: The infant was fed with breast milk, infant formula and/or other types of food.
- Exclusive infant formula feeding: The infant was fed with infant formula, but without any breast milk.

### Study design and sample

The Deyang region is located in the northeast of Chengdu Plain in Sichuan province (southwestern China). In 2011, it had a population of 3.9 million and 36,000 babies were born [19]; 57 % of the people live in rural areas, while the sex ratio (male: female) is 105.2 : 100 [19].

Based on the UNICEF data between 2008 and 2012, 99 % of Chinese women had access to an institutional delivery [13]. In this study, a cross-sectional sampling was used in two urban hospitals and five randomly selected rural clinics in the Deyang region of Southwestern China (Sichuan province). In 2012, 204 mothers with newborns were recruited consecutively on postnatal wards on the basis of informed consent. Interviews were conducted before discharge using a structured questionnaire. The place of origin, urban or rural, was defined as the permanent living area of the participants, but this could have been different from their birthplaces.

### Data collection

The structured questionnaires for women admitted to postnatal wards until discharge included five parts: Part 1 recorded birth outcomes of the newborns and type of delivery; Part 2 assessed the initial infant feeding patterns; Part 3 consisted of mothers’ perceptions about breastfeeding; Part 4 collected the mothers’ anthropometric data (retrospectively) and gestational weight gain, and Part 5 covered the mothers’ socio-demographic data.

### Statistical analysis

Data on maternal characteristics, birth outcomes and breastfeeding practices were described using means (SD) and frequencies. Differences in maternal and newborn characteristics between urban and rural areas were assessed by the independent Student’s *t*-test, Fisher’s exact test or Pearson’s Chi-Square test, as appropriate. Logistic regression was applied to identify factors associated with exclusive/almost exclusive breastfeeding [29]. The following variables were assessed: Caesarean section, preterm birth (babies born alive before 37 weeks of pregnancy), sex of newborn, birth weight, low birth weight, birth length < 50 cm, time to initiating breastfeeding, mother’s knowledge of the importance of colostrum, mother’s attitude about the safety of infant formula, gestational weight gain, and mother’s age, pre-pregnancy Body Mass Index (BMI), education, income, and place of origin (urban vs. rural). The significance level was defined as a *p*-value < 0.05; all statistical analysis was carried out using SPSS software (SPSS Inc., Chicago, IL; Version 20).

### Qualitative study

In addition, in order to explore the social/cultural reasons influencing mothers’ feeding practices, five focus group discussions (FGDs) with three to six local breastfeeding mothers and their infants were held in 2013. On the basis of informed consent, a total of 21 mothers were randomly recruited during their monthly child health care visits at one urban hospital. Questions about awareness of early initiation of breastfeeding, personal experiences of delay of the initiation of breastfeeding and the use of prelacteal food, reasons why the mothers could not breastfeed exclusively, factors that could help mothers to initiate breastfeeding, and reasons for infant formula preference were discussed in the FGDs. The discussions were recorded by digital voice recorder and transcribed onto computer later. NVivo 10 and Microsoft Word 2011 (Mac version) were used to analyze the transcripts. Thematic analysis was applied during the process of creating categories. After transcription and familiarization with the original data, initial codes were generated from the data answering the questions. Similar codes were classified into categories according to their themes. The final categories were reviewed and defined by two researchers. As a supplemental part of the data, results from FGDs were incorporated into the results and discussion part of this paper.

### Ethical considerations

This study conforms to the provision of the 1995 Declaration of Helsinki (revised in Edinburgh, 2000) and was approved by the Medical Committee of Deyang City, China (dated: May 9, 2010).

## Results

In 2012, a total of 204 mothers with newborns (urban *n* = 102, rural *n* = 99, missing value *n* = 3) participated in this study. Five focus group discussions (each with a total 21 mothers) were held in 2013.

### Mothers’ demographic and anthropometric status

Urban mothers gave birth at older ages, were taller, better educated and had a higher mean income than mothers from rural areas (Table 1).

**Table 1:** Demographic and anthropometric characteristics of urban and rural mothers on postnatal wards

	Total	Urban	Rural	*p*-value
	*n* = 201	*n* = 102	*n* = 99	
Age, years	26.0 ± 4.2	26.9 ± 3.7	25.0 ± 4.5	0.001
Weight pre-pregnancy, kg	50.5 ± 6.8	51.1 ± 6.9	49.8 ± 6.7	0.193
Height, m	1.58 ± 0.05	1.59 ± 0.05	1.57 ± 0.05	0.006
BMI ^a^, pre-pregnancy, (kg/m^2^)	20.35 ± 2.65	20.33 ± 2.54	20.36 ± 2.78	0.943
BMI <18.5, n (%)	42 (22.2)	21 (21.9)	21 (22.6)	
BMI 18.5 ~ 24.9, n (%)	137 (72.5)	71 (74.0)	66 (71.0)	
BMI 25 ~ 29.9, n (%)	8 (4.2)	4 (2.1)	4 (4.3)	
BMI ≥30, n (%)	2 (1.1)	0	2 (2.2)	
Parity	1.12 ± 0.33	1.09 ± 0.29	1.16 ± 0.37	0.151
Education				
≤12 years (senior high school), n (%)	141(70.9)	55 (53.9)	86 (88.7)	<0.001
>12 years (i.e. university or above), n (%)	58 (29.1)	47 (46.1)	11 (11.3)	
Monthly income, RMB ^b^	2683 ± 2542	3074 ± 3086	2241 ± 1648	0.026

Values are mean ± SD or number (%)

*p*-value: independent-samples *T* test or Fisher’s exact test

^a^BMI, body mass index (weight/squared height), categorized following WHO standard [35, 61]

^b^Chinese currency, 1 RMB = 0.1622 US Dollar (2014.08.28)

### Birth outcomes

The percentage of urban and rural infants born at term (37–42 weeks, gestational age) was (89.1 % vs. 98.0 %). Premature and postmature births occurred more frequently in urban areas than in rural areas (see Table 2). Urban girls were slightly heavier and taller than rural girls, however, this difference was not significant. By contrast, urban boys were significantly heavier and taller than rural boys. The prevalence of low birth weight (<2.5 kg) was nearly the same in urban and rural areas, whereas high birth weight (≥4 kg) was more common in urban than rural areas (see Table 2).

**Table 2:** Birth outcomes of urban and rural newborns

	Total	Urban	Rural	*p* - value
	*n* = 201	*n* = 102	*n* = 99	
Premature birth (<37 weeks), n (%)	4 (2.0)	4 (4.0)	0	
Term birth (37–42 weeks), n (%)	186 (93.5)	90 (89.1)	96 (98.0)	
Postmature birth (≥42 weeks), n (%)	9 (4.5)	7 (6.9)	2 (2.0)	
Gestational weight gain (GWG) ^b^, kg	15.7 ± 5.3	16.7 ± 5.2	14.6 ± 5.2	0.007
If BMI < 18.5; GWG, kg	15.8 ± 5.5	18.4 ± 5.3	13.1 ± 4.3	
If BMI 18.5 ~ 24.9; GWG, kg	15.8 ± 4.9	16.1 ± 4.7	15.4 ± 5.0	
If BMI ≥ 25; GWG, kg	14.0 ± 9.9	16.3 ± 14.3	12.8 ± 8.4	
Type of delivery				
Vaginal delivery, n (%)	66 (33.0)	35 (34.3)	31 (31.6)	0.764
Caesarean section, n (%)	134 (67.0)	67 (65.7)	67 (68.4)	
Sex of newborns				
Girls, n (%)	119 (59.2)	58 (56.9)	61 (61.6)	0.566
Boys, n (%)	82 (40.8)	44 (43.1)	38 (38.4)	
Birth weight, kg	3.27 ± 0.40	3.32 ± 0.40	3.21 ± 0.39	0.053
Girls (*n* = 119, 58, 61), kg	3.25 ± 0.39	3.27 ± 0.40	3.23 ± 0.37	0.607
Boys (*n* = 82, 44, 38, kg	3.29 ± 0.41	3.39 ± 0.38	3.18 ± 0.42	0.021
Low birth weight < 2.5 kg, n (%)	6 (3.0)	3 (2.9)	3 (3.0)	–
Normal 2.5 ~ 4 kg, n (%)	188 (93.5)	95 (93.1)	93 (93.9)	
High birth weight ≥ 4 kg, n (%)	7 (3.5)	4 (3.9)	3 (3.0)	
Birth length ^a^, cm	49.5 ± 1.5	49.8 ± 1.7	49.2 ± 1.4	0.005
Girls (*n* = 114; 56, 58), cm	49.3 ± 1.4	49.4 ± 1.4	49.1 ± 1.5	0.280
Boys (*n* = 78; 42, 36), cm	49.8 ± 1.7	50.3 ± 1.9	49.2 ± 1.2	0.005
Birth length < 50 cm ^a^, n (%)	79 (41.1)	32 (32.7)	47 (50.0)	0.019

Values are mean ± SD or number (%)

*p* - value: independent-samples *T* test or Fisher’s exact test

^a^ *n* = 98 urban and *n* = 94 rural newborn (total *n* = 192); according to the *Growth standard of Chinese children under 7 years* [62], the median of length of male newborns was 50.4 cm while the female newborns was 49.7 cm. Normally Chinese doctors use *0*–*3 years growth chart* [63] to evaluating the development of the newborns, in which the minimum scale of length is 0.5 cm. In order to simplify the process, 50 cm has become a common cut off point for judgment

^b^Recommendation for weight gain according to pre-pregnancy BMI: Underweight (BMI < 18.5) 12.5–18 kg; Normal range (BMI 18.5–24.9) 11.5–16.0 kg; Overweight/Obese (BMI ≥ 25) 5–11.5 kg [64]

### Infant feeding practice on postnatal wards

The prevalence of different types of infant feeding on postnatal wards is illustrated in Table 3. Soon after birth, nearly all mothers (98.0 % urban vs. 99.0 % rural) started breastfeeding (received breast milk with/without other drink, formula or other infant food). Although only 89.1 % (96.9 % urban vs. 81.1 % rural) of the mothers knew the term “colostrum”, 94.4 % (93.0 % urban vs. 95.8 % rural) of the postpartum mothers stated that their babies received it.

**Table 3:** Type of infant feeding practiced by urban and rural mothers in postnatal wards

	Total	Urban	Rural
	*n* = 200	*n* = 102	*n* = 98
Exclusive breastfeeding, n (%)	16 (8.0)	10 (9.8)	6 (6.1)
Almost exclusive breastfeeding, n (%)	69 (34.5)	30 (29.4)	39 (39.8)
Mixed feeding, n (%)	112 (56.0)	60 (58.8)	52 (53.1)
Exclusive infant formula feeding, n (%)	3 (1.5)	2 (2.0)	1 (1.0)

Only 8.1 % of urban mothers and 5.2 % of rural mothers could meet the requirement for “early initiation of breastfeeding within the first hour of life”. Most of the mothers started breastfeeding at ≥ 2^nd^ day postpartum (Table 4). Overall, results about initiation and onset of breastfeeding were similar among urban and rural mothers (*p* = 0.092).

**Table 4:** Distribution of time to start breastfeeding by urban and rural mothers

	Total	Urban	Rural
	*n* = 196	*n* = 99	*n* = 97
Within 1 h after birth, n (%)	13 (6.6)	8 (8.1)	5 (5.2)
1–6 h after birth, n (%)	27 (13.8)	15 (15.2)	12 (12.4)
7–24 h after birth, n (%)	14 (7.1)	8 (8.1)	6 (6.2)
2nd day after birth, n (%)	54 (27.6)	25 (25.3)	29 (29.9)
3rd day after birth, n (%)	54 (27.6)	20 (20.2)	34 (35.1)
4th day after birth or later, n (%)	34 (17.3)	23 (23.2)	11 (11.3)

### Exclusive/almost exclusive breastfeeding (EBF/AEBF)

Before the initiation of breastfeeding, 87.3 % (86.0 % urban vs. 88.7 % rural) of the newborns were fed with prelacteal food. The most commonly used prelacteal food was infant formula (82.7 % urban vs. 86.5 % rural), followed by warm water (11.2 % urban vs. 10.4 % rural), sugar water (2.0 % urban vs. 1.0 % rural) and fruit juice. One urban baby was given fish liver oil as the first food.

Infant formula was used as a supplement to breast milk on the postnatal wards in 47.9 % (51.5 % urban vs. 44.3 % rural) of infants. Therefore, due to the high prevalence of prelacteal feeding before breastfeeding initiation and the common use of supplementary feeding, the prevalence of exclusive breastfeeding in the postnatal wards was only 8.0 % (Table 3). More urban women (9.8 %) than rural women (6.1 %) exclusively breastfed.

Logistic regression analysis revealed that women who initiated breastfeeding within the first two days (*≤*2 days) were more likely to breastfeed exclusively or almost exclusively (EBF/AEBF). By contrast, factors such as birth length <50 cm, higher educational level (>12 years), knowledge about the importance of colostrum, and Caesarean section reduced the likelihood of EBF/AEBF (Table 5). Caesarean section was also associated with a lower proportion of mothers who initiated breastfeeding within two days (Odds Ratio [OR] 0.489, 95 % Confidence Interval [CI] 0.266, 0.896; *p* = 0.021).

**Table 5:** Factors associated with exclusive (EBF) and/or almost exclusive breastfeeding (AEBF) in postnatal wards

Factors	OR	95 % CI	*p-* value
Breastfeeding initiated ≤ 2 days	1.97	(1.11–3.50)	0.020
Birth length of newborns < 50 cm	0.48	(0.26–0.87)	0.016
Mother’s education > 12 years	0.46	(0.24–0.88)	0.019
Mothers have knowledge about the importance of colostrum	0.35	(0.14–0.86)	0.023
Caesarean section	0.55	(0.31–1.00)	0.051

*OR* odds ratio, *CI* confidence interval, for EBF and/or AEBF

To understand the reasons for widespread delay of the initiation of breastfeeding and the low prevalence of exclusive breastfeeding as well as the high use of infant formula FGDs were conducted (Tables 6 and 7). Many mothers had never heard about starting breastfeeding within one hour of birth, and a number of mothers believed that the secretion of breast milk wouldn’t start immediately after birth. Several mothers explained that after a Caesarean section, it would be difficult to breastfeed comfortably because of pain at the site of the incision. Furthermore, nipple problems were also believed to be one of obstacles to early initiation of breastfeeding (Table 6). Regarding exclusive breastfeeding, first and foremost, insufficient and inadequate knowledge, and also external influences, such as societal expectations and the pressure of advertising were found to be the major factors. Altogether, these reasons contributed to a mother’s feeling of inadequacy. Many women doubt their capability to nourish their babies, and others fear that breastfeeding is harmful for the shape of their breasts (Table 7).

**Table 6:** The reasons for delay of initiation of breastfeeding given by mothers in Focus Group Discussions

Classified reasons	Representative quotes
Lack of knowledge about early initiation of breastfeeding	• *“The doctors and nurses didn’t tell us to start breastfeeding immediately (after childbirth)…The grandparents also didn’t mention that.”*
No breast milk at the beginning	• *“Immediately after birth we had no idea that there would be some breast milk available soon after delivery”* • *“I could not see/feel any breast milk after delivery. Therefore, there was no milk ready to be sucked out.”*
Caesarean section	• *“Because I had a Caesarean section, the wound was still painful after birth. Therefore, I started to breastfeed my baby on the third day after delivery”* • *“After the Caesarean section, we expressed a bit of colostrum for the baby by hand”*
Large nipples	• *“The baby refused to suck, because the nipples were too big for him. About three days later, he was ready to start sucking.”*

**Table 7:** Reasons for low prevalence of exclusive breastfeeding and preference of infant formula mentioned by mothers in the Focus Group Discussions (*n* = 21)

Classified reasons	Representative quotes
Knowledge about EBF	• *“Many mothers never heard about exclusive breastfeeding.”*
Amount of breast milk is not enough	• *“We know breast milk is the best, but we were afraid that the amount is not enough. We don’t know when the baby is full.”* • *“Because sometimes, if the breast milk is not enough, the baby will cry, so we give him infant formula in addition.”* • *“I am afraid that my breast milk is not enough and consequently my baby will not grow/develop properly.”*
	• *“I have no idea. I want to breastfeed exclusively, but the amount of my breast milk is not enough.”*
	• *“Many of my friends in my generation didn’t have breast milk after childbirth at all.”*
	• *“In fact, most of the mothers are willing to breastfeed their babies because breast milk is the best. But there is no way to breastfeed exclusively, because their breast milk is not enough.”*
Breast milk is not nutritious enough (influence of advertising)	• *“Probably the nutrient content in my milk is not enough, therefore I use infant formula as supplement.”*
	• *“Because infant formula has many advantages, it is rich in some nutrients like DHA (docosahexaenoic acid), taurine that are good for baby’s development. But the content of these nutrients is low in mothers’ milk.”*
	• *“Infant formula preference is often influenced by advertising for it everywhere.”*
Necessary to add water	• *“Someone with experience told us water is necessary besides breast milk.”*
Parents’ concerns	• *“As we only have one child, all parents cherish their babies and worry too much that their baby may get hungry.”*
	• *“Everyone treasures the baby so much and thinks that with infant formula the baby will develop better. Many mothers believe that ‘infant formula is necessary in addition to breast milk.’”* • *“The feeling that “all the other babies are fed with infant formula” makes mothers think it is wrong if they don’t give infant formula to their own babies. Mothers are afraid of the development of their babies falling behind others.”*
Mother’s figure	• *“Mothers choose infant formula instead of breastfeeding for the shape of their breasts; it’s a common problem. Anyway,I don’t care about my figure, I will insist on breastfeeding.”*
	• *“At present, mothers have different perceptions. Some mothers do not want to breastfeed, just like my friend who believes breastfeeding is harmful for her figure.”*
Convenience	• *“Some mothers are using infant formula for convenience.”*
	• *“The babies fed with infant formula will not be hungry easily. Their mothers do not need to wake up and feed the babies so frequently in the night. It’s more convenient.”*
Wound pain after C/S	• *“It’s painful after Caesarean section. It may influence exclusive breastfeeding (hard to hold the baby).”*

## Discussion

### Disparities between urban and rural areas

Over the past two decades, China has made substantial progress in maternal health [20], and the gap between urban and rural maternal mortality is closing. In 1991, there were 46 urban vs. 100 rural maternal deaths per 100.000 live births and in 2012, 22 urban vs. 26 rural respectively [30]. However, the National Maternal Mortality Surveillance System in China (NMMSS) reported that between 2001 and 2005 the risk ratio of preventable maternal mortality in rural areas had doubled compared to urban areas (OR 2.38, 95 % CI 2.01, 2.81) [31]. According to the results of this study, some of the disparities in social, demographic and anthropometric status between urban and rural mothers still existed in the study area. On average, urban mothers had significantly higher incomes, were better educated and were about two years older when they conceived than were rural mothers (see Table 1). One study in rural western China indicated that the age of the woman was positively correlated with anemia and that the higher the wealth index of the household, the lower the prevalence of maternal anemia [32]. NMMSS reported that, in remote rural areas, preventable maternal mortality accounted for 97 % of maternal deaths, while the most frequent factors were associated with mothers’ lack of adequate knowledge [31].

Referring to the mothers’ anthropometric status before pregnancy, in this study, urban mothers were two centimeters taller than rural mothers. Potentially, this may have influence on birth outcomes, the type of delivery, as well as the type of infant feeding, all of which are discussed later in this paper. Most of the mothers in both areas had a pre pregnancy BMI within the normal range. Slightly more mothers tended to be underweight or overweight/obese in rural compared to urban areas. This study found that the proportion of underweight mothers was high in both urban and rural areas (21.9 % urban vs. 22.6 % rural), while the prevalence of overweight/obesity was relatively low in both areas (2.1 % urban vs. 6.5 % rural). This finding is in line with the results from another cross-sectional study in the same region [33], and with a study conducted in northeastern parts of China by Liu et al. [34]. By contrast, Pei et al. [35], found a relatively low prevalence of maternal underweight (7.9 %) and a comparably higher prevalence of overweight/obesity (17.9 %) in rural Sichuan province.

With respect to birth outcomes, urban newborns were significantly taller and heavier than rural infants in the study area, which could be explained by the fact that the urban mothers were taller and gained more weight during pregnancy than rural mothers. The prevalence of low birth weight in the study area was 3 %, which was consistent with the Chinese national level [13], with no significant difference between urban and rural areas. Although the problems of low birth weight and high birth weight infants were not serious in the study area, since pre pregnancy underweight is known to be associated with poor birth outcomes (i.e., premature birth, low birth weight) [34], more attention needs to be paid to urban and rural underweight women of reproductive age, especially for rural underweight pregnant women as recommended by Gao et al. [33].

We found nearly all mothers started breastfeeding (98.0 % urban vs. 99.0 % rural) in the Deyang region within the first five days after birth. Similar results were found by Qiu et al., any breastfeeding being practiced substantially in both city (96.5 %) and rural areas (97.4 %) [36], by Tang et al. (93.5 % rural) [25] and by Guo et al. (98.3 % in total) [9]. However, with reference to exclusive breastfeeding before discharge, the prevalence of EBF was extremely low in both urban (8.1 %) and rural areas (5.2 %), as compared to a hospital-based study by Qiu et al. (38.0 % urban vs. 61.0 % rural) [36]. Although in this study, no significant difference in infant feeding in urban and rural areas was found on the postnatal wards, the incidence was inadequate in both settings (see Table 5). It is clear, therefore, that there must be other factors impacting mothers’ infant feeding practices, an issue that will be discussed below.

### Initiation of breastfeeding

In this study, only 6.6 % of the mothers initiated breastfeeding within one hour of birth. This finding was slightly lower, but similar to the results by Tang et al. (9.1 %) [27] and Lou et al. (11.1 %) [37] in the same region of Sichuan province. This is in line with the fact that local people in Sichuan lack the knowledge (only 26.5 % undergraduates agreed) that “breastfeeding should be started within the first hours after birth” [38]. However, when looking at the results of a broader regional study [9], 6.6 % was far behind the 59.4 % level in central and western China.

“Why did the mothers delay the initiation of breastfeeding?” was a question discussed in the FGDs (see Table 6). A “lack of knowledge about early initiation of breastfeeding” and “Caesarean section” was given as the main reasons. Both quantitative and qualitative results reflected that: (a) there is a need to improve the implementation of the “Ten Steps to Successful Breastfeeding” [39], both in urban hospitals and rural clinics in the study area and (b) it could be explained by a misunderstanding of what “initiate breastfeeding” means in the local area. In the study area, “initiate breastfeeding” does not have the same meaning as “start sucking”. The mothers insisted they would not start breastfeeding until they felt that they had breast milk or that their breasts felt engorged, even if the baby had started sucking (breastfeeding) several hours or even a few days earlier. This misunderstanding may partly explain why the study results for EBF were so low as well.

### Exclusive breastfeeding

Due to the widespread delay in breastfeeding, prelacteal feeding and the use of infant formula as a supplement to breast milk, the prevalence of EBF was only 8 % on postnatal wards. This situation was worse than the results of Chinese regional studies 28.7 % [9], 24.2 % [8] and at the national level 28.0 % [13]. Without prelacteal feeding, the prevalence of EBF/AEBF could be up to 42.5 % in this study.

When the newborns’ lengths were shorter than 50 cm, their chances of being breastfed (almost) exclusively were lower compared to those who were ≥ 50 cm. In other words, if the mother had borne a smaller sized baby, her confidence in being able to breastfeed exclusively was reduced. Based on these findings, nurses and midwives should offer more breastfeeding support to the mothers with smaller sized infants, because these infants could benefit very significantly from the higher bioavailability of nutrients in breast milk compared to infant formula [40, 41].

In this study, the time to initiate breastfeeding ≤ two days after delivery was also significantly correlated with EBF/AEBF. The earlier the mothers started breastfeeding, the more likely they were to breastfeed exclusively. Although Caesarean section (CS) was found to be associated with EBF/AEBF, indirectly it was related to the time of initiating breastfeeding. Many other studies noted that CS delays the initiation of breastfeeding [42, 43] and is associated with the use of supplements among newborns [44] and mothers who had CS experienced more breastfeeding problems [45, 46]. Similar to earlier findings in the same study area [47], the proportion of CS was over 60 % during the study period and CS could defined as one of the risk factors for not exclusively breastfeeding. Thus, further research to reduce the incidence of CS in China would be important.

Unexpectedly, both the mothers’ educational levels and their knowledge of colostrum were negatively correlated with their feeding practices. Those findings were similar to some study results in other countries [48–50]. In focus group discussions, the mothers’ knowledge of the definition of exclusive breastfeeding was found to be poor. Several mothers had no idea what exclusive breastfeeding meant (Table 7) or knew the optimal time of exclusive breastfeeding. Other quotes included misinformation; “besides breast milk, water is necessary” and “exclusive breastfeeding is good, but difficult to practice”.

The FGDs revealed a common misunderstanding that “there was no/too little breast milk available immediately after childbirth” (Table 7). This perception was reported by mothers, grandparents, peers, and even some of the medical staff; many expectant mothers had purchased a box of infant formula and brought it to the hospital prior to giving birth. This may help to explain why more than 80 % of the infants received infant formula as a prelacteal feed. This misunderstanding was also related to the delay in the initiation of breastfeeding, as the mothers believed that there was nothing in the breasts at the beginning, when the breasts felt soft, and therefore they needed to wait until they were engorged or when they felt they had started to have breast milk. In addition, the belief that “water is essential as the first drink for newborns” was frequently mentioned by mothers in focus group discussions. This misunderstanding needs to be addressed during local breastfeeding promotion/education sessions.

Mothers tried to explain the low prevalence of exclusive breastfeeding in China during the FGDs (Table 7) and the feeling that “the amount of breast milk produced was not enough” was one of the major reasons mentioned. In other studies, “insufficient breast milk syndrome” has also been considered an important barrier to successful breastfeeding [37, 51, 52]. Because mothers usually cannot measure the amount of breast milk a baby has drunk, the mothers in our study often lacked confidence about whether their babies were satisfied or wondered if they were still hungry. In order to match the requirements of the growth charts at the monthly child care examination, these mothers preferred giving additional infant formula to their babies after breastfeeding. In most cases, monitoring the amount and color of the urine in the diaper and the frequency of bowel movements is a more effective indicator than mothers’ feelings [37]. Daily weighing of the infant in the hospital and every couple of days after discharge could reassure the mothers and improve their confidence about exclusive breastfeeding. Due to the fact that most of the mothers delayed the initiation of breastfeeding, prelacteal feedings were widely used. Supplementation was continued after initiating breastfeeding because of the concern of not producing enough milk. During the course of this study, it became obvious that the physiology of breast milk production was not clearly understood. Thus, education on the physiology of breast milk production and secretion and the related importance of frequent suckling should be included in the maternal school curriculum.

Due to the widespread inappropriate advertising for infant formula in China [10, 53], parents believed that many health benefits are linked to formula feeding. Perceptions, such as infant formulas being as nutritious or even superior to breast milk, influenced several mothers to offer these formulas as supplements in addition to breastfeeding. To reduce the impact of the unsubstantiated health claims of the infant formula companies [54] the Ministry of Health of China issued a ban on aggressive advertising of infant formula in 1995 [55]. However, due to lack of control mechanisms, the ban does not seem to have been effective.

Another barrier to exclusive breastfeeding in China is the shifting attitude about breasts among modern young people. Historically, the main function of breasts was simply ‘feeding’. People now perceive “small and exquisite breasts” as beautiful [56]. According to traditional Chinese culture, small feet not breasts, were the important sexual organs [56–58]. However, in contemporary China, the sexual role of breasts is surpassing their feeding function, just as in other industrialized countries [57, 59, 60]. More and more mothers and their husbands choose infant formulas instead of breastfeeding, because they believe breastfeeding could cause mothers’ breasts to shrivel or sag. To address this increasing problem, mothers need a better understanding of the physical structure and the function of breasts through improved breastfeeding education. The mainstream media could take responsibility for highlighting maternal aspects of breastfeeding rather than focusing only on the erogenous function of breasts.

### Limitations

It is likely that women, who delivered their babies vaginally, without any complications, were discharged earlier and, consequently, were not included in our postnatal assessment which took place during the first week after delivery. Therefore, women who delivered their newborns by Caesarean section may be overrepresented by comparison to those who delivered without any complications and who left the hospital earlier. In addition, the traditionally dominant roles of family members (i.e. father, grandmother) were not adequately considered in this study with regard to infant feeding recommendations and this could have been useful in explaining the breastfeeding situation in the study area.

## Conclusion

Although disparities in infant feeding between urban and rural areas exist, breastfeeding rates are inadequate in both settings. The extremely low prevalence of exclusive breastfeeding among newborns was found to be associated with delayed initiation of breastfeeding and widespread use of infant formula in addition to breastfeeding. Directly and indirectly; the high prevalence (67 %) of Caesarean section, the mothers’ poor knowledge of the physiology of breast milk production, the aggressive advertising of infant formula as well as the shifting perception of the primary function of the breasts by modern people have led to a unfavorable breastfeeding status in the study area. At hospital level, the implementation of the “Ten Steps to Successful Breastfeeding” could be strengthened in both urban and rural areas. Nationally, effective systems for monitoring the inappropriate advertising of infant formula are necessary. At the same time, the mainstream media should also take responsibility for breastfeeding promotion. Furthermore, exploring the reasons for high Caesarean section rates in the local area and methods of reduction would be another important research field.

## Abbreviations

WHO: World Health Organization
FGDs: focus group discussions
BMI: body mass index
GWG: gestational weight gain
EBF: exclusive breastfeeding
AEBF: almost exclusive breastfeeding
NMMSS: National Maternal Mortality Surveillance System in China
